# Dietary intake in healthy older individuals is associated with lipopolysaccharide binding protein a biomarker of gut function: an exploratory cross-sectional study

**DOI:** 10.3389/fragi.2025.1572867

**Published:** 2025-03-31

**Authors:** Debra Jones, Douglas J. Morrison, Stuart R. Gray, Susan E. Ozanne, Carlos Celis-Morales, Mahek Jain, Lewis R. Mattin, Matthew Gittins, Saleh A. A. Alkhedhairi, James L. Dorling, Sorrel Burden

**Affiliations:** ^1^ School of Health Sciences, University of Manchester, Manchester, United Kingdom; ^2^ Scottish Universities Environmental Research Centre (SUERC), University of Glasgow, Glasgow, United Kingdom; ^3^ School of Cardiovascular and Metabolic Health, University of Glasgow, Glasgow, United Kingdom; ^4^ Institute of Sports Science and Innovation, Lithuanian Sports University, Kaunas, Lithuania; ^5^ Institute of Metabolic Science - Metabolic Research Laboratories and MRC Metabolic Diseases Unit, University of Cambridge, Addenbrookes Hospital Cambridge, Cambridge, United Kingdom; ^6^ School of Life Sciences, University of Westminster, London, United Kingdom; ^7^ Department of Medical Biosciences, College of Veterinary Medicine, Qassim University, Buraidah, Saudi Arabia; ^8^ Human Nutrition, School of Medicine, Dentistry and Nursing, College of Medical, Veterinary and Life Sciences, University of Glasgow, Glasgow, United Kingdom; ^9^ Salford Care Organisation, Northern Care Alliance NHS Trust, Salford, United Kingdom

**Keywords:** healthy ageing, gut health, nutrient intake, fibre intake, physical function, gut function biomarkers, lipopolysaccharide binding protein, LBP

## Abstract

Diet, physical function and gut health are important modifiable factors in ageing. However, it is unclear how ageing affects various domains of gut function. Aims of this cross-sectional study were to explore relationships between nutrient intake, physical function, and biomarkers of gut function in older individuals. Healthy participants (n = 94, mean age 71.1 years SD 5.10, 56% female) were recruited to investigate the relationship between nutrient intake (protein, fibre, carbohydrate, fat), physical function (chair rise time, handgrip strength) and lipopolysaccharide (LPS) binding protein (LBP); a marker of gut permeability. Linear regression models, adjusted for age, fat mass/fat free mass ratio, weight and gender, reported LBP changed by; −161.9 ng/mL (95% CI -323.0, −0.8) for every 1 g increase in daily fibre/1,000 kilocalories; 80.5 ng/mL (6.7, 154.2) for 1% increase in daily energy intake as fat; and −88.1 ng/mL (−146.7, −29.6) for 1% increase in daily energy as carbohydrates. When further adjusted for C-reactive protein (CRP), a marker of inflammation, LBP decreased by an additional 6.9 ng/mL for fibre, increased by an additional 4.0 ng/mL for fat and decreased by an additional 3.7 ng/mL for carbohydrate. These findings suggest that in healthy older adults’ nutrient intake is associated with LBP, and CRP appears to slightly modify these associations. There were no associations between LBP and handgrip strength or chair rise time. Results suggest that fibre, fat, and carbohydrates are important for maintaining gut function, potentially mediated by inflammation in older adults, although further research is needed to explore the implications for physical function and CRP as a mediator.

## 1 Introduction

The complex process of ageing involves a series of physiological changes that lead to increased vulnerability and susceptibility to diseases. This has been broadly defined as a time-dependent decline in physical and mental functions necessary for maintaining health and survival (Lopez-Otin et al., 2013; Madeira et al., 2021). Except for the setback observed after 2019, due to COVID-19, international trends indicate people are living longer (World Health Organization, 2024). Increased life expectancy represents an important health challenge as a longer lifespan does not necessarily mean a longer healthspan (WHO, 2019). Recent findings from a recent United Kingdom government report stated that increases in health expectancies are not in keeping with gains in life expectancy, particularly in older adults (Foresight: Government Office for Science, 2015).

Appropriate nutrition and prevention of malnutrition plays a crucial role in supporting the healthy ageing process with decreased risk of age-related chronic diseases and mortality being associated with diets low in ultra-processed foods and rich in lean proteins, wholegrains, fruits, vegetables and healthy fats (Dominguez et al., 2024). Nutrient intake also impacts substantially on gut health (Sanders et al., 2023; Xu and Knight, 2015),with dietary fibre (Cronin et al., 2021; Fu et al., 2022; Koh et al., 2016), polyphenols (Cardona et al., 2013; Hussain et al., 2016; Mithul Aravind et al., 2021; Ross et al., 2024), and omega-3 fatty acids (Costantini et al., 2017) established as important components in the maintenance of a healthy digestive system. These nutrients are detected by the gut through a process known as gut nutrient sensing, where enteroendocrine cells release hormones in response to the presence of nutrients (Gribble and Reimann, 2022). These hormones are responsible for regulating some important gut functions such as gut motility and downstream processes like appetite regulation and insulin secretion (Steinert et al., 2017), linking nutrient intake directly to gut health and metabolic response. As a result, gut health can directly impact overall health and the ageing process by influencing immune function (Wiertsema et al., 2021); development of non-communicable diseases such as inflammatory bowel disease and obesity (Hou et al., 2022); onset of age related conditions including frailty, musculoskeletal diseases and metabolic and neurological disorders (Ghosh et al., 2022; Salazar et al., 2023); and the ability to be physical active (Valdes et al., 2018). Additionally, reduction in the diversity of the gut microbiota has been linked with increased inflammation, frailty and metabolic diseases (Donati Zeppa et al., 2022; O'Toole and Jeffery, 2018). Consequently, maintaining a healthy digestive system and gut microbiota into older age is essential for achieving disease free and healthy life years in later life.

When the gut epithelium is compromised, cell and bacterial components can translocate from the gut into the bloodstream. Some of these components are termed gut biomarkers as they can be measured in the blood to provide an indication of gut health (Hoshiko et al., 2021; Meng et al., 2021; Perez-Diaz-Del-Campo et al., 2023; Seethaler et al., 2021). Lipopolysaccharide (LPS), a large molecule in the outer membrane of Gram-negative bacteria (Meng et al., 2021), can “leak” through the intestinal epithelium indicating increased bacterial translocation and increased gut permeability or “leaky gut.” Unfortunately, measuring LPS is challenging and complicated, therefore, lipopolysaccharide binding protein (LBP) and Cluster of differentiation 14 (CD14), which are part of the inflammatory signalling pathway of LPS, are considered useful alternatives to LPS as markers of gut translocation (Mogilevski et al., 2024a). In addition Fatty Acid Binding Protein 2 (FABP2), which is found in the intestinal epithelium and plays a role in the transportation of fatty acids, complements LBP and CD14 as a gut biomarker as it is prone to leak into the bloodstream when the intestinal cell wall is damaged (Mogilevski et al., 2024b).

Monitoring novel gut biomarkers, such as LBP, CD14, and FABP2 can provide valuable insights into the impact of nutrient intake on gut health in the adult population (Aleman et al., 2023; Camilleri, 2021; Seethaler et al., 2021; Usuda et al., 2021). For example, a study measuring dietary intake in 129 healthy participants (<50 years) found an inverse association between fibre intake and plasma LBP. (Bailey et al., 2023). Similarly, a systematic review including 10 studies conducted in patients with type II diabetes concluded that fibre intake significantly decreased LPS, although effect on LBP was not significant (Ojo et al., 2021). Healthy dietary patterns have also been associated with lower levels of LPB or LPS. For instance, Fuke et al. (2023) conducted a large cross-sectional study in 896 healthy adults in Japan and concluded that LBP correlated negatively with intake of healthy foods and nutrients including vegetables and dietary fibre (Fuke et al., 2023). Likewise, a large prospective study of 912 patients with atrial fibrillation demonstrated that LPS levels were negatively affected by a high adherence to the Mediterranean diet (Pastori et al., 2017). Therefore, previous work has demonstrated that in the healthy adult population and in certain disease states there is an association between healthy dietary intake and gut biomarkers, such as LBP. However, there is a paucity of studies in this area for the healthy older adult population and it is still unclear if these novel gut biomarkers can be used in older people for promotion of healthy ageing by gauging the impact of diet on gut health.

In this pilot study we compiled information from multiple data streams to create a unique dataset from an aged cohort, to consider the associations between gut nutrient sensing and ageing. The aim was to combine LBP, CD14 and FABP2 gut biomarker data with phenotypic data and explore the relationship between reported nutrient intake, physical function and gut health prevalence in a group of older individuals. The null hypothesis was that nutrient intake and inflammation in older people is not associated with LBP, CD14 or FABP2 gut function biomarkers.

## 2 Materials and methods

### 2.1 Study design and population

This study followed The Strengthening the Reporting of Observational Studies in Epidemiology (STROBE) guidelines and checklist for cross-sectional studies (von Elm et al., 2008), see Supplementary Material (SI) Table 1. The study design was a cross-sectional secondary data analysis study that used retrospective phenotypic data and blood samples collected from older individuals participating in a previous study (Alkhedhairi et al., 2022). Recruitment for the original study took place between March 2018 and March 2020 from the Glasgow area and sample size was based upon the primary outcome of knee extensor maximal strength. Using a standard deviation (SD) of 9% (based on data from the lab of the original study) a sample size of 50 participants per group was calculated (80% power at P < 0.05) and aimed to recruit 120 to account for dropouts. The study was approved by the University of Glasgow Medical, Veterinary, Life Sciences College Research Ethics Committee [Reference 200170067] and was carried out in accordance with the ethical standards established in the 1964 Declaration of Helsinki. Participants were randomised to either control or krill oil supplementation.

**TABLE 1:** Characteristics of healthy older participants.

Variable	N	Healthy older adults
Age, mean (SD) (yrs)	94	71.1 (5.0)
Gender, n (%) Male Female	94	41 (43.6) 53 (56.4)
Anthropometrics, mean (SD) Height (m) Weight (kg) BMI (kg/m^2^) Body fat mass (%) FM/FFM ratio (kg)	94	1.7 (0.1) 71.8 (12.3) 25.7 (3.4) 30.4 (7.8) 0.5 (0.2)
Physical function, mean (SD) Handgrip strength (kg) Chair rise time (seconds) Within chair rise cut off of 12.8 s, n (%)	94	31.3 (9.1) 11.9 (1.2) 69.0 (73.4)
Protein intake Mean (SD) (g) Protein intake as % of energy intake (%) As a % of protein DRV (%) Meets protein DRV, n (%)	94	69.1 (15.9) 17.1 (3.0) 140.2 (32.2) 85.0 (90.4)
Fibre intake, mean (SD) AOAC fibre (g) AOAC fibre intake per 1000 kcal (g) Meets AOAC 30 g/d recommendation, n (%) NSP fibre (g) NSP fibre intake per 1000 kcal (g) Meets NSP 18 g/d recommendation, n (%)	94	7.1 (3.1) 4.3 (2.5) 0.0 (0) 13.7 (4.8) 8.5 (2.8) 15.0 (16.0)
Other nutritional intake, mean (SD) Energy intake (kcal) Meets energy intake recommendation, n (%) Fat intake (g) Fat intake as % of energy intake (%) Saturated fat intake (g) Carbohydrate intake (g) Carbohydrate intake as % of energy intake (%) Total vegetable intake (g) Total fruit intake (g) Total fruit and vegetable intake (g) Portions of fruit and veg per day (1 portion = 80 g) Meets 5 a day fruit and veg, n (%)	94	1,650.0 (392.7) 11 (11.7) 67.3 (23.0) 36.2 (5.7) 24.9 (9.0) 191.6 (49.2) 46.7 (7.1) 177.6 (78.4) 189.3 (122.2) 366.9 (153.9) 4.6 (1.9) 47.0 (50.0)
CRP Mean (SD) (mg/mL) Low CRP (<1.0 mg/mL), n (%) Medium to high CRP (≥1.0 mg/mL), n (%)	93	1.5 (1.5) 49.0 (52.7) 44.0 (47.3)
Gut biomarkers, mean (SD) Lipopolysaccharide binding protein (ng/mL) Cluster of differentiation 14 (ng/mL) Fatty acid binding protein 2 (pg/mL)	90	5,874.9 (2006.9) 1,525.3 (267.5) 1798.3 (661.7)

N/n; number, SD: standard deviation, yrs: years, %: percentage, m: meters, BMI: body mass index, FMI: fat mass index, g: grams, kg: kilograms, kg/m2: kilograms per meters squared, DRV: dietary reference value, AOAC: association of analytical chemists, kcal: kilocalories, g/d: grams per day, NSP: non-starch polysaccharides, CRP: C-reactive protein, mg/mL: milligrams per millilitre, ng/mL: nanograms per millilitre, pg/mL: picograms per millilitre, veg: vegetable.

For this study we used the previous data collected at baseline including participant characteristics, weight, height, body mass index (BMI), body fat, muscle mass, handgrip strength, chair rise time test, nutritional intake (2-day dietary recall). In addition, we utilised the blood samples collected at baseline and carried out further analysis to ascertain levels of gut function biomarkers. Sample size for this study was based on the sample collected from the original study.

Older adults aged 65 years and older, participating in less than 1 hour of self-reported exercise per week and with a BMI less than 35 kg/m2 were enrolled into the original study. Exclusion criteria were reported co-morbidities including diabetes mellitus, severe cardiovascular disease, seizure disorders, uncontrolled hypertension, active cancer or cancer in remission within the previous 5 years, ambulatory impairments (which would limit ability to perform assessments of muscle function), and dementia. Additional participants were excluded if they were taking medication known to affect muscle, having an implanted electronic device, on anticoagulant therapy or nutritional supplements, allergic to seafood or regular consumption of more than two portions of oily fish per week. All participants included in the original study were eligible for this study.

The exposures of interest for this study were nutrient intake, including energy, protein, fibre, carbohydrate, and fat, and inflammation measured using C reactive protein. The outcome for this study was gut function measured using LBP, CD14 and FABP2, with higher levels of LBP, CD14 and FABP2 considered to be indicative of reduced gut barrier integrity. Key data collected on participant characteristics, body composition and physical function were also utilised for this study.

As this was a cross sectional it may have been susceptible to bias, including selection bias and recall bias. To address these potential sources of bias the study was advertised in the community using posters and newspaper and magazine adverts with the aim of random recruitment from a wide population. Dietary intake data was collected using dietary recall over a 2-day period, with an average of the 2 days reported.

### 2.2 Dietary intake and body composition assessments

In the original study habitual dietary intake data were collected using an online software (myfood24.org) for multi-pass (dietary recall is enquired about multiple times at different levels of detail) 2-day dietary recalls with an average of the 2 days reported. Body composition was measured using bioelectrical impedance analysis with a TANITA-DC-430MAS, specifically for the measurements of muscle mass and body fat percentage.

For this study nutrient intake across individuals was calculated as a percentage of energy intake for protein, carbohydrate, fat, and for the Association of Analytical Chemists (AOAC) fibre and non-starch polysaccharide (NSP) intake in grams was expressed per 1,000 kcal. For adequate energy intake we calculated the percentage of participants meeting the United Kingdom government dietary recommendation for older adults of 1,912 kcal per day and 2,342 kcal per day for women and men respectively who are 65–74 years of age (Scientific Advisory Committee on Nutrition, 2021). For adequate protein intake we calculated the percentage of participants meeting the United Kingdom government dietary recommendation for older adults of 53.3 g and 46.5 g of daily protein intake for women and men respectively who are 65 years and over (Scientific Advisory Committee on Nutrition, 2021). For adequate fibre intake we calculated the percentage of participants meeting the dietary reference values (DRV) for adults of 30 g of daily AOAC fibre intake and 18 g of daily NSP fibre intake (Scientific Advisory Committee on Nutrition, 2015). For adequate physical function we calculated the percentage of participants meeting the recommended cutoff values of less than 16 kg for women and 27 kg for men for weak handgrip strength (Dodds et al., 2014) and under 12.8 s for inadequate chair rise time (Kim and Won, 2022). To assess participants level of inflammation we calculated the percentage of participants with C-reactive protein (CRP) levels under 1.0 mg/mL for low inflammation and CRP levels of 1.0 mg/mL and above for medium to high inflammation (Nehring et al., 2024). For appropriate representation of body composition, we calculated fat mass/fat free mass ratio [kg] (FM/FFM). FM [kg] was converted from body fat percentage [(weight [kg] * fat%)/100] and FFM [kg] was calculated by subtracting FM [kg] from body weight.

### 2.3 Laboratory procedures

Plasma samples taken during the original study period were stored at 80°C and later accessed for use in this study. Samples for all 94 included participants were sent to NIHR Core Biochemistry Assay Laboratory, Cambridge Biomedical Research Centre for analysis. LPS binding protein was measured using the electrochemiluminescence immunoassay R-plex Human LBP (lipopolysaccharide-binding protein) assay (catalogue number K151K5R-2) from MesoScale Discovery (Rockville, MD, United States). The assay was performed according to the manufacturer’s instructions with the samples diluted 500-fold prior to the analysis. Soluble CD14 and iFABP were measured using the quantitative sandwich enzyme linked immunosorbent assay Quantikine^®^ assay from Bio-Techne R&D Systems (Abingdon, Oxford) (soluble CD14 product code DC140; iFABP (Human FABP2/I-FABP) product code DFBP20). Both assays were performed according to the manufacturer’s instructions.

### 2.4 Statistical analysis

Data analysis was carried out using Stata statistical analysis software (StataCorp LP, version 14.0). Participant characteristics were summarised and reported using standard descriptive statistics. Gut biomarkers and CRP inflammatory markers were assessed for their distributional properties and spurious results. All gut biomarkers and CRP were considered to have a normal distribution, and any spurious results were corrected within 5% and 95% of the sample (Ruppert, 2006). The primary analysis looked at the associations of nutrient intakes and energy, with gut function. A secondary exploratory analysis was also performed including CRP as a covariate in the model. This explored whether CRP is a potential mediator of any relationships. Linear regression models were used to investigate the association between each of the gut biomarker outcomes (LBP, CD14 and FABP) and exposure to each nutrient (protein, fibre, fat, carbohydrates, energy, fruit and vegetables) and exposure to having adequate physical function (hand grip strength and chair rise time). Models were initially run without adjustment (model 1), then with adjustment (model 2) for age, gender (male/female), FM/FFM ratio and weight. FM/FFM ratio was used for adjustment rather than BMI as FM/FFM ratio may better classify those at higher risk of mortality and functional decline, particularly in older people where functional decline is more likely to be present (Cruz-Jentoft et al., 2019; Jensen et al., 2019; Merchant et al., 2021; Xiao et al., 2018). An additional analysis further adjusted for CRP to investigate whether the nutrient effect on gut function biomarkers was modified (model 3). CRP was included here as a covariate in the final model to explore the possibility of CRP as a potential mediator. Assumptions of linearity were met for the regression models. Post hoc tests were completed for each regression analysis revealing normal distribution of residuals, quantiles and linear prediction. See Supplementary Figure S1.

## 3 Results

### 3.1 Baseline characteristics of participants

Complete data was available for this study from 94 participants of the original study. Participants were aged 65–85 years old, just over half were female (56.4%) and mean FM/FFM ratio was 0.5 kg (SD 0.2). In terms of physical function most participants (99.0%) met the recommended handgrip strength cutoff for adequate muscle strength and just under three-quarters of participants (73.4%) met the required 12.8 s chair rise time for sufficient physical function. Participants mean daily nutrient intakes included energy intake of 1,650.0 kcal (SD 392.7), protein intake of 69.1 g (SD 15.9), AOAC fibre intake of 7.1 g (SD 3.1) and NSP fibre intake of 13.7 g (SD 4.8). Daily intake of protein, total fat and carbohydrate as a mean percentage of energy intake was 17.1% (SD 3.0), 36.2% (SD 5.7), and 46.7% (SD 7.1) respectively. Daily intake of AOAC fibre and NSP fibre per 1,000 kcal was 4.3 g (SD 2.5) and 8.5 g (SD 2.8) respectively. Most participants (90.4%) met the recommended DRV for protein intake in older adults (65 years and over), however, less than a fifth of participants (16.0%) met the DVR for NSP fibre, 11.7% met the recommended energy intake for older people, and none of the participants met the DRV for AOAC fibre. Inflammatory CRP was recorded in 93 out of the 94 participants with a mean of 1.5 mg/mL and just over half of participants (52.7%) considered to have a low CRP (<1.0 mg/mL). Gut function biomarkers were recorded in 90 out of the 94 participants. Further detailed participant characteristics are displayed in Table 1.

### 3.2 Association of nutrient intake and physical function with gut function biomarkers

**FIGURE 1:**
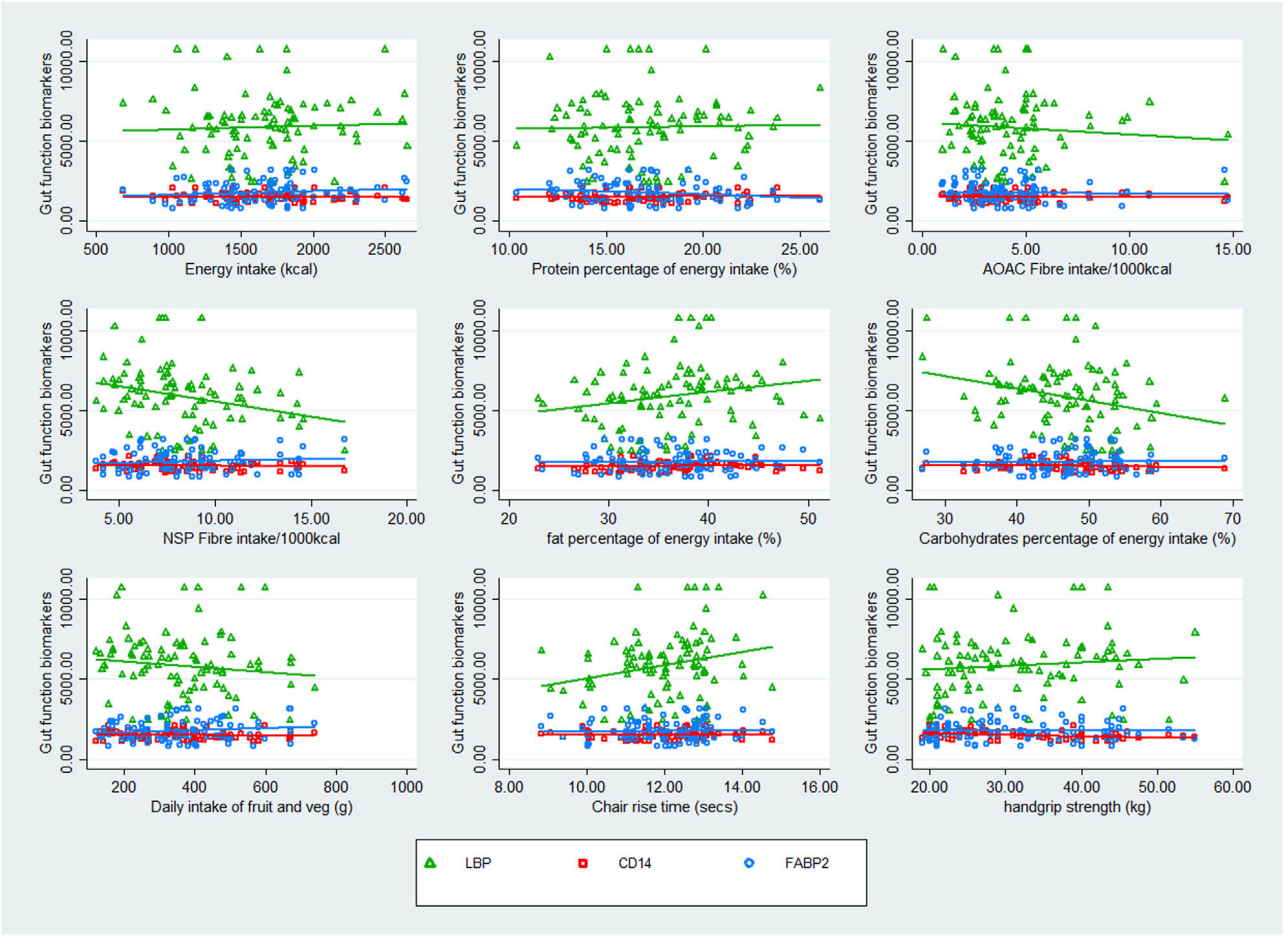
Scatter plots of nutrient intake and physical function in association with markers of gut function.

Scatter plots crudely describing the association between nutrient intake and the three gut function biomarkers suggested that there was no clear association between CD14 or FABP2 and any of the recorded nutrients and physical function tests. However, there was a negative correlation between LBP and AOAC fibre, NSP, carbohydrate, fruit and vegetable intake, and handgrip strength, and a positive correlation between fat intake and chair rise time. See Figure 1.

Table 2 reports effect estimates and 95% Confidence Intervals (95% C.I) of the linear regression models of LBP gut function biomarker due to the intake of certain nutrients and levels of physical function (model 1). The adjusted model indicated that daily protein intake, AOAC fibre intake, fruit and vegetable intake, chair rise time and handgrip strength were not associated with LBP (model 2). However, daily intake of NSP fibre, fat, and carbohydrates were associated with LBP. Linear regression models, when adjusted for age, FM/FFM, weight and gender reported LBP changed by; −161.9 ng/mL (95% CI -323.0, −0.8) for every 1 g increase in daily fibre/1,000 kilocalories; 80.5 ng/mL (6.7, 154.2) for 1% increase in daily energy intake as fat; and −88.1 ng/mL (−146.7, −29.6) for 1% increase in daily energy as carbohydrates. See Table 2.

**TABLE 2:** Linear regression analysis to explore dietary intake and physical function in association with LBP gut function biomarker.

	LBP gut biomarker (ng/mL), n = 90					
	Model 1 unadjusted		Model 2 adjusted		Model 3 adjusted	
Variable	β	95% CI	β	95% CI	β	95% CI
Energy intake (kcal)	0.2	−0.9 to 1.3	0.2	−0.9 to 1.4	0.2	−0.9 to 1.3
Protein as % of daily energy intake	17.4	−120.3 to 155.1	12.65	−126.7 to 152.0	23.9	−112.9 to 160.6
AOAC fibre per 1000 kcal (g)	−79.5	−251.6 to 92.7	−82.4	−255.4 to 90.49	−93.3	−262.6 to 76.0
NSP fibre per 1000 kcal (g)	−190.3	−340.4 to −40.2*	−161.9	−323.0 to −0.8*	−168.8	−326.1 to −11.6*
Fat as % of daily energy intake	69.5	−3.1 to 142.2	80.5	6.7 to 154.2*	84.5	12.6 to 156.5*
Carb as % of daily energy intake	−70.7	−134.7 to −20.6*	−88.1	−146.7 to −29.6*	−92.4	−149.4 to −35.4*
Daily fruit and veg intake (g)	−1.7	−4.7 to 1.2	−0.5	−3.8 to 2.8	−0.6	−3.8 to 2.6
Chair rise time (seconds)	413.8	60.4 to 767.2*	308.3	−70.5 to 687.0	297.0	−74.1 to 668.0
Handgrip strength (kg)	20.9	−25.09 to 66.9	−11.6	−86.0 to 62.8	3.77	−70.5 to 78.1

LBP: lipopolysaccharide binding protein, ng/mL: nanograms per millilitre, β:Beta coefficient, 95% CI: 95% confidence intervals, g: grams, %: percentage, DRV: dietary reference value, AOAC: association of analytical chemists, kcal: kilocalories, NSP: non-starch polysaccharides, carb: carbohydrates, veg: vegetables, kg: kilograms *: *p*<0.05.

Supplementary Tables S2, S3 report effect estimates and 95% C.I. of the linear regression models of the CD14 and FABP2 gut function biomarkers due to the intake of certain nutrients and levels of physical function. After adjusting for age, FM/FFM, weight and gender these regression models showed no associations with any of the variables being investigated.

### 3.3 Exploratory investigation of CRP inflammatory marker on the association of nutrient intake or physical function with gut function biomarkers

**FIGURE 2:**
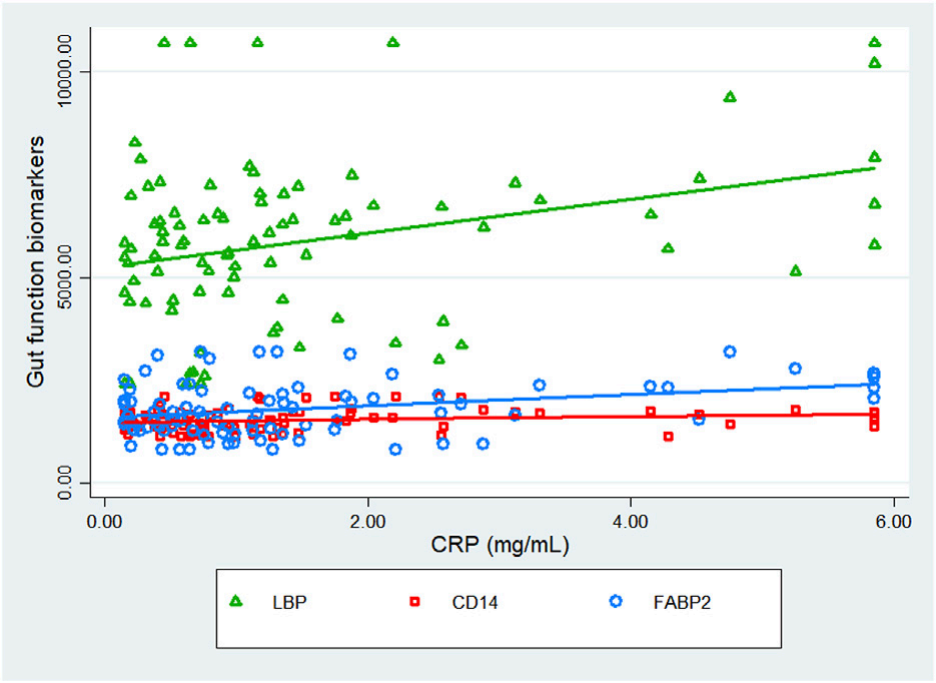
Scatter plot of inflammatory marker CRP in association with gut function biomarkers.

Scatter plots crudely describing the association between CRP and gut function biomarkers suggested a positive correlation with LBP and no correlation or very little correlation with CD14 and FABP2. See Figure 2. Model 3 of the regression analysis was designed to assess the impact of medium to high CRP on nutrient intake and physical function in association with LBP. Model 3 of the regression analysis revealed no association of daily intake of protein, AOAC fibre, NSP fibre and chair rise time with LBP, when further adjusted for CRP, a theoretical mediator. However, model 3 did indicate an association of NSP fibre, fat and carbohydrate intake with LBP, when further adjusted for CRP. Model 3 demonstrated that in comparison to model 2 LBP decreased by an additional 6.9 ng/mL for every 1 g higher in daily intake of NSP fibre per 1,000 kcal, increased by an additional 4.0 ng/mL for every percentage higher in daily energy intake due to fat and decreased by an additional 3.7 ng/mL for every percentage higher in daily energy intake due to carbohydrates. See Table 2.

## 4 Discussion

This study uniquely investigates the associations of diet and physical function on LBP, CD14 and FABP2 gut function biomarkers in healthy older individuals. Although there were no associations with physical function, the study found that dietary fibre, carbohydrates and fat were associated with LBP. Participants reporting a higher dietary fibre and carbohydrate intake were also exhibiting lower LBP levels, suggesting they had adequate gut barrier integrity, whereas those reporting greater fat intake had higher LBP levels, suggesting poorer gut barrier integrity, indicating possible dysbiosis. Furthermore, accounting for CRP appeared to result in a small increased association between nutrient intake associations with LBP, suggesting that inflammation may have potential mediating effects on these relationships. Although, caution is advised as the lack of association with handgrip strength and chair rise time suggests that LBP may not be a suitable marker for physical function in older people. Additionally, the change in association when adjusted for CRP was relatively small, which may mean that CRP would fail to have any mediating effects in a study with a longitudinal design.

These findings align with existing research that healthy dietary patterns are negatively associated with levels of LBP in the blood and so could be used as a strategy to reduce systemic inflammation and metabolic disease. Recent large cross sectional and prospective studies have shown that intake of fibre (Bailey et al., 2023; Ojo et al., 2021) and the consumption of healthy dietary patterns (Fuke et al., 2023; Pastori et al., 2017) are negative associated with LBP or LPS in both healthy and diseased adult populations. Interesting, a recent randomised control trial conducted over 12 months reported that a decrease in the faecal proportion of palmitoleic acid was associated with adherence to the Mediterranean diet and decreased LBP (Seethaler et al., 2024). Indeed, high fat, Western-style diets have been linked to increased gut permeability and systemic inflammation (Brown et al., 2012; Claesson et al., 2012; Kang et al., 2023), whereas diets high in fibre, and Mediterranean-style diets are linked to maintaining gut barrier function (Abrignani et al., 2024; Gundogdu and Nalbantoglu, 2023; Nagpal et al., 2019; Valdes et al., 2018).

Dietary fibre is considered vital in maintaining gut health (Cronin et al., 2021; Fu et al., 2022) and adherence to healthy dietary patterns such as the Mediterranean diet, rich in fresh fruits, vegetables, legumes, fibre, polyphenols and monounsaturated fatty acids, has been associated with beneficial patterns of probiotic taxa in the gut microbiome (Gundogdu and Nalbantoglu, 2023; Jones et al., 2024). This type of diet promotes the release of gut hormones that augment satiety and maintain gut barrier function (Abrignani et al., 2024; Nagpal et al., 2019). Conversely, unhealthy diets such as the Western diet, high in processed foods, sugars and saturated fats can lead to dysbiosis, a state of microbial imbalance associated with poorer metabolic health (Claesson et al., 2012; Kang et al., 2023). Therefore, this study adds to the evidence base that poor diet quality, particularly high fat and low fibre, can disrupt gut nutrient sensing processes, leading to increased gut permeability and systemic inflammation, (Brown et al., 2012). Furthermore, this ideais explored in older adults, a population that has been less studied in this context.

In contrast, this study also demonstrates that nutrient intake and physical function in older adults was not associated with CD14 and FABP2. This could indicate that whilst these markers of gut function are relevant in some contexts, they may not be as responsive to changes in nutrient intake or physical function in an older, relatively healthy population. Indeed, CD14 is associated with inflammatory immune responses as a marker of monocyte activation (Sharygin et al., 2023; Shive et al., 2015) and FABP2 (also known as intestinal fatty-acid binding protein) is a marker of epithelial damage to the gut (Hoffmanova et al., 2015; Lau et al., 2016). Most participants in this study met the cutoffs for adequate physical function and the mean CRP level was within normal range, both of which may explain the lack of association found with CD14 and FABP2. It is possible that these biomarkers may be more relevant in populations compromised by physical functional or metabolic diseases or with higher levels of systemic inflammation. Indeed, half of the sample in this study had low levels of CRP, indicating a low level of systemic inflammation, which could be supporting overall health and providing protection against age-related diseases.

In this study NSP fibre intake had the strongest association with LBP, however, most of the participants did not meet the recommended intake for dietary fibre (Scientific Advisory Committee on Nutrition, 2015) or recommended energy intake for older people, despite having adequate physical function and meeting the DRV for protein intake (Scientific Advisory Committee on Nutrition, 2021). Although, there may be a degree of under reporting, leading to lower reported levels of energy and fibre intake. However, low energy and fibre intake in older adults could be an area of concern, especially given the importance of fibre to gut health and could well be an important factor to target for prevention of deterioration in older, otherwise healthy individuals.

The results of this study may help support public health policy that aims to improve healthspan by increasing disease free and healthy years in later life (Office for Health Improvement and Disparities, 2023; WHO, 2019). This work may also help in addressing some of the malnutrition research priorities raised in a recent James Lind Alliance Priority Setting Partnership (Jones et al., 2020) as well as further highlighting the importance of dietary interventions in mitigating age-related decline by supporting gut health and function.

### 4.1 Strengths and limitations

The even gender split, a mean age of 71.10 years, a mean BMI under 30 kg/m2 and a mean chair rise time under 12.8 s indicates that the cohort used in this study generally represented a healthy older population. However, the small sample size (n = 94) could limit the generalisability of the findings. The use of a 2-day food frequency questionnaire to record dietary intake may not be representative of actual intake, compared to a 3-day diary, including a weekend day. In addition, this study analysed singular nutrients rather than dietary patterns, which may overlook the impact of whole diet on gut function. Equally, there was a lack of nutrient breakdown including intake of fatty acid type, grain type and sugars, which could limit the ability to identify the influence of specific nutrients on gut health. It is also important to note that this study was secondary data analysis for hypothesis generating. In addition, a cross-sectional study is not suitable for measuring causal inference or for implementing mediation analysis as the cross-sectional data is a snapshot in time so does not contain the requisite temporal component. Therefore, the results demonstrating associations and from the analysis relating to CRP as a potential mediator should be read with caution as further longitudinal investigation is required to establish causal links. It is also important to note that the intestinal barrier can be influenced by many other factors, which this study was not able to account for, including stress, psychological stress, disease, alcohol, antibiotics, drug consumption and exercise (Aleman et al., 2023; Camilleri, 2021; Mogilevski et al., 2024b). Any future work should be designed with these factors in mind. Nonetheless, the results achieved here are based on data from a trial, with well-designed and controlled data collection, and overall provides valuable insights into the impact of diet and physical function in community dwelling healthy older individuals.

## 5 Conclusion

The findings of this study suggest nutrient intake in older adults, particularly dietary fibre intake, is associated with gut function as indicated by LBP levels. Given the association of greater fibre intake on lower LBP levels, and the low numbers of participants meeting fibre intake recommendations, future research should further explore the long-term effects of dietary fibre and other dietary patterns and components in gut health, amongst large and diverse older adult populations. Furthermore, CRP appears to moderate the association between nutrient intake and LBP, although the effect is very small, nevertheless the potential of CRP as a mediator should be explored. Since diet and physical function on CD14 and FABP2 did not show any associations, it may be beneficial to investigate alternative biomarkers with better sensitivity to dietary changes or physical function in community dwelling, relatively healthy older adults. These results highlight the importance of high fibre diets for maintaining gut health and the need for longitudinal randomised control trials to explore the impact of nutrient intake, nutrient sensing and gut health in ageing populations.

## Data Availability

The raw data supporting the conclusions of this article will be made available by the authors, without undue reservation.
