# Supplementary material for: Dietary intake in healthy older individuals is associated with lipopolysaccharide binding protein a biomarker of gut function: an exploratory cross-sectional study
Source: Front Aging. 2025 Mar 31;6:1572867. doi: 10.3389/fragi.2025.1572867 (PMC11994966; doi:10.3389/fragi.2025.1572867)
Supplement: Supplementary file 1 [file DataSheet1.docx]

Supplementary Material

# Supplementary Table 1

**STROBE Statement—Checklist of items that should be included in reports of *cross-sectional studies***

|  | Item No | Recommendation | Page number |
| --- | --- | --- | --- |
| **Title and abstract** | 1 | (*a*) Indicate the study’s design with a commonly used term in the title or the abstract |  |
|  |  | (*b*) Provide in the abstract an informative and balanced summary of what was done and what was found |  |
| Introduction | | |  |
| Background/rationale | 2 | Explain the scientific background and rationale for the investigation being reported |  |
| Objectives | 3 | State specific objectives, including any prespecified hypotheses |  |
| Methods | | |  |
| Study design | 4 | Present key elements of study design early in the paper |  |
| Setting | 5 | Describe the setting, locations, and relevant dates, including periods of recruitment, exposure, follow-up, and data collection |  |
| Participants | 6 | (*a*) Give the eligibility criteria, and the sources and methods of selection of participants |  |
| Variables | 7 | Clearly define all outcomes, exposures, predictors, potential confounders, and effect modifiers. Give diagnostic criteria, if applicable |  |
| Data sources/ measurement | 8* | For each variable of interest, give sources of data and details of methods of assessment (measurement). Describe comparability of assessment methods if there is more than one group |  |
| Bias | 9 | Describe any efforts to address potential sources of bias |  |
| Study size | 10 | Explain how the study size was arrived at |  |
| Quantitative variables | 11 | Explain how quantitative variables were handled in the analyses. If applicable, describe which groupings were chosen and why |  |
| Statistical methods | 12 | (*a*) Describe all statistical methods, including those used to control for confounding |  |
|  |  | (*b*) Describe any methods used to examine subgroups and interactions |  |
|  |  | (*c*) Explain how missing data were addressed |  |
|  |  | (*d*) If applicable, describe analytical methods taking account of sampling strategy |  |
|  |  | (*e*) Describe any sensitivity analyses |  |
| Results | | |  |
| Participants | 13* | (a) Report numbers of individuals at each stage of study—eg numbers potentially eligible, examined for eligibility, confirmed eligible, included in the study, completing follow-up, and analysed |  |
|  |  | (b) Give reasons for non-participation at each stage |  |
|  |  | (c) Consider use of a flow diagram |  |
| Descriptive data | 14* | (a) Give characteristics of study participants (eg demographic, clinical, social) and information on exposures and potential confounders |  |
|  |  | (b) Indicate number of participants with missing data for each variable of interest |  |
| Outcome data | 15* | Report numbers of outcome events or summary measures |  |
| Main results | 16 | (*a*) Give unadjusted estimates and, if applicable, confounder-adjusted estimates and their precision (eg, 95% confidence interval). Make clear which confounders were adjusted for and why they were included |  |
|  |  | (*b*) Report category boundaries when continuous variables were categorized |  |
|  |  | (*c*) If relevant, consider translating estimates of relative risk into absolute risk for a meaningful time period |  |
| Other analyses | 17 | Report other analyses done—eg analyses of subgroups and interactions, and sensitivity analyses |  |
| Discussion | | |  |
| Key results | 18 | Summarise key results with reference to study objectives |  |
| Limitations | 19 | Discuss limitations of the study, taking into account sources of potential bias or imprecision. Discuss both direction and magnitude of any potential bias |  |
| Interpretation | 20 | Give a cautious overall interpretation of results considering objectives, limitations, multiplicity of analyses, results from similar studies, and other relevant evidence |  |
| Generalisability | 21 | Discuss the generalisability (external validity) of the study results |  |
| Other information | | |  |
| Funding | 22 | Give the source of funding and the role of the funders for the present study and, if applicable, for the original study on which the present article is based |  |

# Supplementary Figure 1

*LBP: lipopolysaccharide binding protein*

**A:** Post-hoc residuals histogram, quantile normal plot and scatter plot for the adjusted model 2 effect of protein as a percentage of energy intake on LBP

*LBP: lipopolysaccharide binding protein, AOAC: Association of Official Agricultural Chemists*

**B:** Post-hoc residuals histogram, quantile normal plot and scatter plot for the adjusted model 2 effect of AOAC fibre per 1000 kilocalories on LBP

*LBP: lipopolysaccharide binding protein, NSP: non-starch polysaccharide*

**C:** Post-hoc residuals histogram, quantile normal plot and scatter plot for the adjusted model 2 effect of NSP fibre per 1000 kilocalories on LBP

*LBP: lipopolysaccharide binding protein*

**D:** Post-hoc residuals histogram, quantile normal plot and scatter plot for the adjusted model 2 effect of fat as a percentage of daily energy intake on LBP

*LBP: lipopolysaccharide binding protein*

**E:** Post-hoc residuals histogram, quantile normal plot and scatter plot for the adjusted model 2 effect of carbohydrate as a percentage of daily energy intake on LBP

*LBP: lipopolysaccharide binding protein*

**F:** Post-hoc residuals histogram, quantile normal plot and scatter plot for the adjusted model 2 effect of daily fruit & veg intake on LBP

**

*LBP: lipopolysaccharide binding protein*

**G:** Post-hoc residuals histogram, quantile normal plot and scatter plot for the adjusted model 2 effect of chair rise time in seconds on LBP

*LBP: lipopolysaccharide binding protein*

**H:** Post-hoc residuals histogram, quantile normal plot and scatter plot for the adjusted model 2 effect of handgrip strength on LBP

# Supplementary Table 2

**Linear regression analysis to explore the association between dietary intake and CD14 gut biomarker**

|  | **CD14 gut biomarker (ng/mL), n=90** | | | |
| --- | --- | --- | --- | --- |
|  | **Model 1 unadjusted** | | **Model 2 adjusted** | |
| **Variable** | **β** | **95% CI** | **β** | **95% CI** |
| Energy intake (kcal) | -0.1 | -0.2 to 0.1 | >0.1 | -0.1 to 0.2 |
| Protein as % of daily energy intake | 7.8 | -10.5 to 26.1 | 1.6 | -15.6 to 18.9 |
| AOAC fibre per 1000kcal (g) | -0.7 | -23.7 to 22.4 | -4.2 | -25.7 to 17.3 |
| NSP fibre per 1000kcal (g) | -2.3 | -23.1 to 18.37 | -9.8 | -30.1 to 10.5 |
| Fat as % of daily energy intake | 4.9 | -4.9 to 14.7 | 5.6 | -3.7 to 14.9 |
| Carb as % of daily energy intake | -4.1 | -12.0 to 3.8 | -5.0 | -12.5 to 2.6 |
| Daily fruit and veg intake (g) | -0.1 | -0.5 to 0.3 | -0.1 | -0.6 to 0.3 |
| Chair rise time (secs) | 0.2 | -48.4 to 48.7 | -1.0 | -48.6 to 46.6 |
| Handgrip strength (kg) | -8.1 | -14.0 to -2.2* | 3.2 | -5.9 to 12.4 |

**: p<0.05, CD14: Cluster of differentiation 14, ng/mL: nanograms per millilitre, β:Beta coefficient, 95% CI: 95% confidence intervals, kcal: kilocalories, g: grams, %: percentage, DRV: dietary reference value, AOAC: Association of Analytical Chemists, kcal: kilocalories, NSP: non-starch polysaccharides, carb: carbohydrates, veg: vegetables*

# Supplementary Table 3

**Linear regression analysis to explore the association between dietary intake and FABP2 gut biomarker**

|  | **FABP2 gut biomarker (pg/mL), n=90** | | | |
| --- | --- | --- | --- | --- |
|  | **Model 1 unadjusted** | | **Model 2 adjusted** | |
| **Variable** | **β** | **95% CI** | **β** | **95% CI** |
| Energy intake (kcal) | 0.2 | -0.1 to 0.6 | 0.2 | -0.2 to 0.6 |
| Protein as % of daily energy intake | -37.3 | -82.1 to 7.4 | -33.0 | -78.8 to 12.8 |
| AOAC fibre per 1000kcal (g) | -13.0 | -70.0 to 43.9 | -4.2 | -62.0 to 53.6 |
| NSP fibre per 1000kcal (g) | 20.9 | -30.2 to 71.9 | 27.0 | -27.5 to 81.5 |
| Fat as % of daily energy intake | 3.9 | -20.5 to 28.4 | 7.4 | -17.7 to 32.6 |
| Carb as % of daily energy intake | 3.9 | -15.6 to 23.5 | 1.0 | -19.5 to 21.5 |
| Daily fruit and veg intake (g) | 0.7 | -0.3 to 1.7 | 0.5 | -0.6 to 1.6 |
| Chair rise time (secs) | 18.7 | -101.3 to 138.7 | 17.4 | -110.4 to 145.2 |
| Handgrip strength (kg) | -0.8 | -16.1 to 14.4 | 1.4 | -23.3 to 26.2 |

**: p<0.05, FABP2: Fatty acid binding protein 2, pg/mL: picograms per millilitre, β:Beta coefficient, 95% CI: 95% confidence intervals, kcal: kilocalories, g: grams, %: percentage, DRV: dietary reference value, AOAC: Association of Analytical Chemists, kcal: kilocalories, NSP: non-starch polysaccharides, carb: carbohydrates, veg: vegetables*
